# The Structure of Neuronal Calcium Sensor-1 in Solution Revealed by Molecular Dynamics Simulations

**DOI:** 10.1371/journal.pone.0074383

**Published:** 2013-09-30

**Authors:** Luca Bellucci, Stefano Corni, Rosa Di Felice, Emanuele Paci

**Affiliations:** 1 Center S3, CNR Institute Nanoscience, Modena, Italy; 2 Astbury Centre for Structural Molecular Biology, University of Leeds, Leeds, United Kingdom; University of Oldenburg, Germany

## Abstract

Neuronal calcium sensor-1 (NCS-1) is a protein able to trigger signal transduction processes by binding a large number of substrates and re-shaping its structure depending on the environmental conditions. The X-ray crystal structure of the unmyristoilated NCS-1 shows a large solvent-exposed hydrophobic crevice (HC); this HC is partially occupied by the C-terminal tail and thus elusive to the surrounding solvent. We studied the native state of NCS-1 by performing room temperature molecular dynamics (MD) simulations starting from the crystal and the solution structures. We observe relaxation to a state independent of the initial structure, in which the C-terminal tail occupies the HC. We suggest that the C-terminal tail shields the HC binding pocket and modulates the affinity of NCS-1 for its natural targets. By analyzing the topology and nature of the inter-residue potential energy, we provide a compelling description of the interaction network that determines the three-dimensional organization of NCS-1.

## Introduction

Neuronal calcium sensor (NCS) proteins are a conserved subclass of the calmodulin superfamily that triggers different biological processes to regulate signal transduction in neurons and photoreceptor cells [1]–[5]. Among the NCS proteins, the Neuronal calcium sensor-1 (also called frequenin) is expressed essentially in the neuronal cell types. NCS-1 has been implicated in several physiological functions including the regulation of neurotransmission, the synaptic plasticity and learning, the membrane traffic and the activity of ion channels [1]–[3], [5]–[8]. Because NCS-1 is implicated in neuronal regulation, its dysfunctions are directly involved in mental conditions, such as bipolar disorder, schizophrenia and autism [6], [9]–[13].

**Figure 1:**
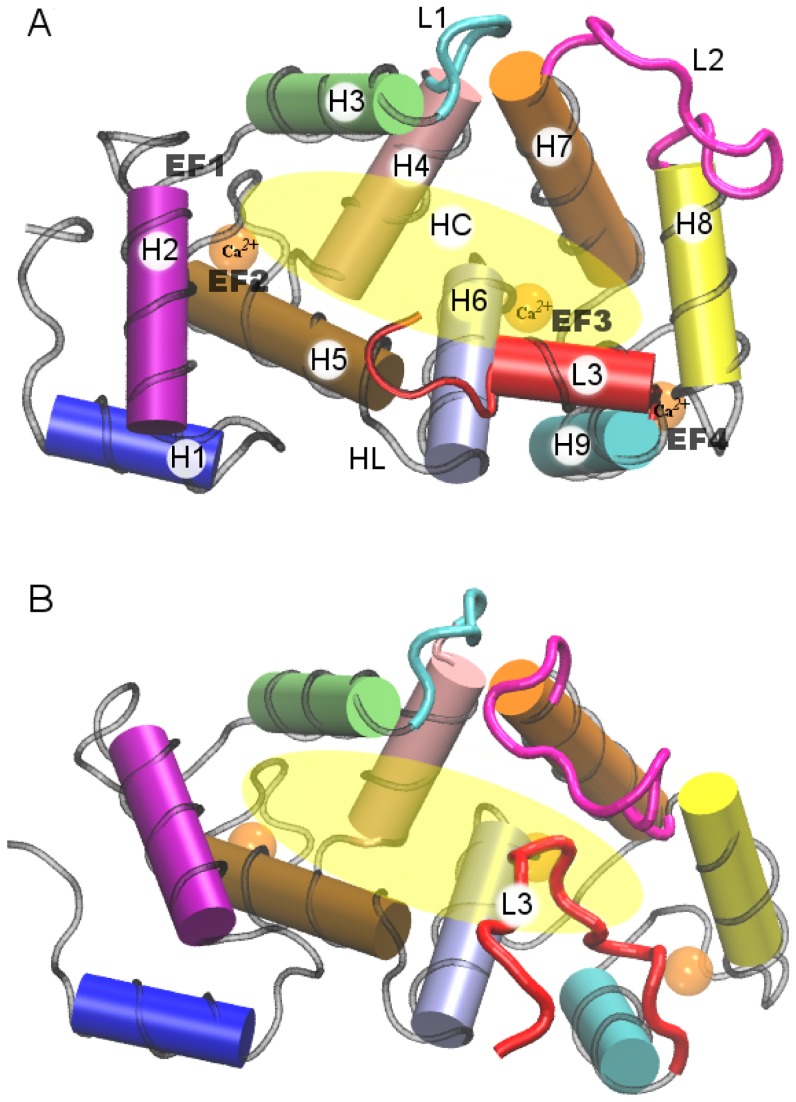
Cartoon representation of the crystallographic and NMR structures. (A) Crystallographic structure from PDB file 1G8I; (B) NMR structure, the first model that appears in the PDB file 2LCP. Residues 11–174 between H1 and H9 define the protein core (PC). The definitions of the other protein segments are given in the section “Material and Method” and in Table 1.

The structure of NCS-1 is characterized by helix-loop-helix motifs, called EF-hand motifs, which can bind Ca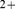 ions. There are 4 EF-hand motifs (Figure 1), namely EF1, EF2, EF3 and EF4, but EF-1 is not able to bind Ca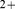 ions [3], [4]. The N-terminal glycine is myristoylated in vivo through the action of N-miristoyltransferase. It is widely accepted that the miristoyl chain confers the capability to the NCS family proteins to link to the membrane through the “myristoyl switching” mechanism, [3], [5], [14], [15] which, eventually, can be modulated by changing the Ca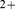 concentration [16]. In the opposite position of the EF-motifs there is a central and wide hydrophobic crevice (HC), that can interact with various physiological targets activating specific biological processes [2], [6], [7], [16]–[18].

Recently, Heidarsson *et al.* [19] determined the NMR structure of the non-myristoylated calcium-bound NCS-1. The solution structure shows that the N-domain is more flexible than the C-domain, and in the absence of a binding partner the hydrophobic C-terminal residues partially occupy the HC.

The NMR experiments, therefore, suggest that the C-terminal segment of NCS-1 may dock into the hydrophobic crevice and act as a ligand-mimic in the absence of an interaction partner, conferring conformational stability to the NCS-1 structure. The structure proposed by Heidarsson *et al.* is free from polyethylene glycol (PEG) molecules and not constrained by crystal packing interactions. Note, however, that the resolution of the C-terminal region is rather poor, compromised by the small number of NMR signals available in this region [19].

In the crystal structure of NCS-1 [4], the C-terminal tail is instead outside the crevice, suggesting a poor affinity between the C-terminal tail and the HC. In this structure the HC is occupied by PEG molecules, mimicking the position of the C-terminal tail observed in the NMR structure and likely inhibiting the binding of the C-terminal tail in the HC.

To investigate the origin of the differences between the solution and crystal structures and to clarify the biological role of the C-terminal, long molecular dynamics simulations in explicit solvent were performed for the non-myristoylated Ca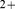-bound NCS-1. Specifically, we carried out two simulations: (i) one starting from the NMR structure and (ii) the other starting from the protein crystal structure modeled without the PEG molecules docked in the HC.

During both simulations the C-terminal tail docked into the HC in a very similar manner. This, in turn, suggests that the C-terminal can dock into the HC acting as an auto-inhibitor, by blocking substrate access to the HC and conferring stability to the protein by shielding the HC from the environment.

To find the critical interactions that stabilize the protein structure, we evaluated the inter-residue interaction potential energy maps including all pairs of residues. The analysis of the inter-residue maps highlights the presence of several salt bridges that interconnect the secondary structures, thus modulating the stability of the NCS-1 tertiary structure [20]–[22]. Based on this discovery, we suggest a way to modify the stability of the NCS-1 by mutating few crucial residues.

## Results

### Substantial differences exist between X-ray and NMR structures

The crystal and solution structures are shown in Figure 1. We carried out a quantitative comparison of the two structures by evaluating total and partial root mean square deviations (RMSDs). This analysis reveals striking differences between the two structures.

The main difference concerns the conformation of the L3 segment. In the X-ray structure, (Figure 1A) L3 is in helical conformation and outside the HC. In the NMR structure, (Figure 1B) L3 is partially docked into the HC. Appreciable differences also involve the relative orientation of the “external” H1, H2, H8 and H9 helices. The RMSD evaluated for the backbone of residues 11 to 174, the protein core (PC), of the NMR structure with respect to the crystallographic structure is 4.4 Å, which reflects substantial divergence. Partial RMSDs for the loops, 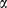-helices and 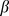-strands between the crystal and solution structures are given in the supplementary information (see Table S1).

Both structures are affected by measurement conditions and may deviate from the inherent protein conformation. On one hand, the X-ray static structure is affected by the presence of glycol molecules [4] and by crystal packing. On the other hand, the NMR solution structure has a poor resolution in the C-terminal segment, because of a small number of NOE signals [19].

### Molecular dynamics simulations converge to a unique state

The MD technique has been assessed as a reliable tool to refine experimental structures [23]–[26]. Thus, two long MD simulations at the temperature of 310 K were performed starting from the X-ray structure (MD-XR) and the NMR structure (MD-NMR).

**Figure 2:**
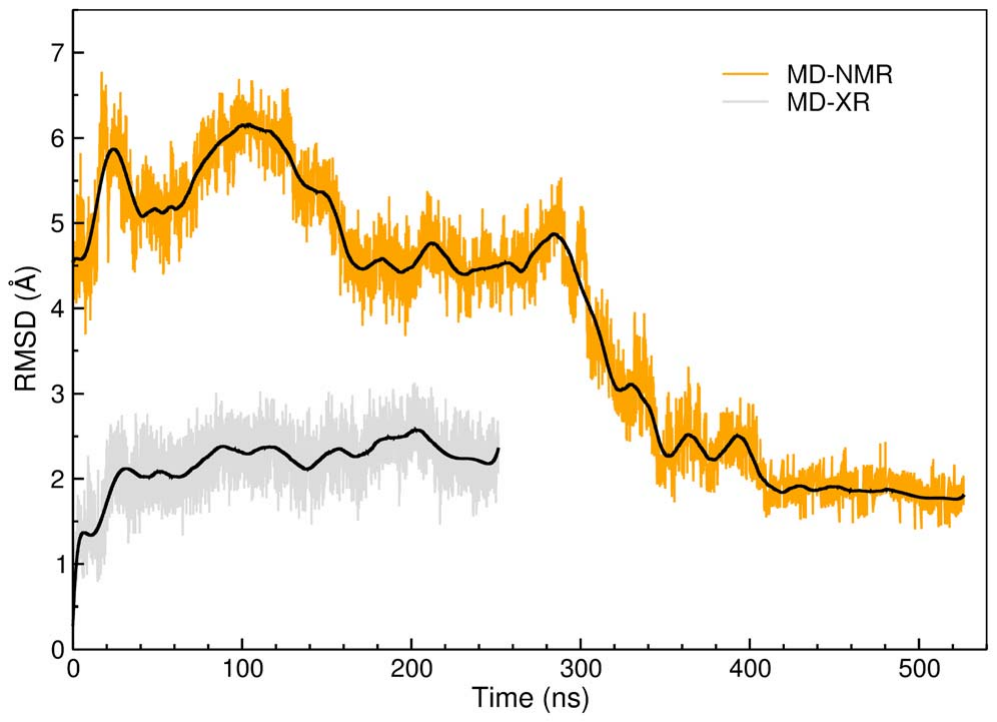
RMSDs of the protein core evaluated with respect to the crystal structure along the MD trajectories. Gray line: RMSD evaluated along the 250 ns of the MD-XR trajectory. Orange: RMSD evaluated along the 525 ns of the MD-NMR simulation. Smoothed RMSDs signals are reported as black lines.

The structural adjustments of the protein during the simulations were monitored by evaluating the RMSD of backbone residues 11 to 174, i.e. the PC, with respect to the original X-ray crystallographic structure. The RMSD values calculated along the MD-XR and MD-NMR simulations are reported in Figure 2 (gray and orange curves, respectively). The RMSD during the MD-XR simulation reaches a plateau after 20 ns and remains stable until the end of the simulation (250 ns). The RMSD does not exceed 3 Å and fluctuates roughly between 2 Å and 2.5 Å. These are evidences that the PC did not undergo substantial conformational rearrangements during the MD-XR simulation (see Figure S1 and Table S2 for more details).

**Figure 3:**
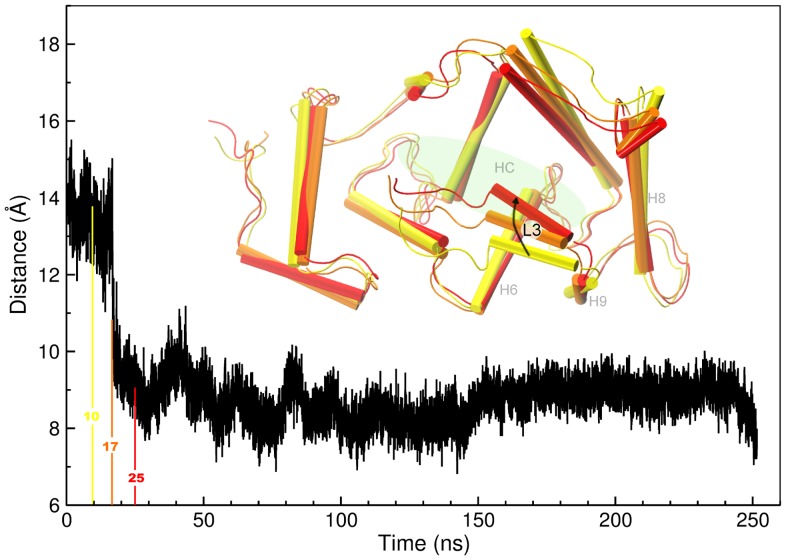
Distance between the COM of the L3 tail and the COM of the whole protein. The inset shows significant snapshots taken at 10, 17 and 25-XR simulation: these snapshots highlight the process of L3 docking.

Nevertheless, during the first 20 ns of the MD-XR simulation L3 docked into the HC and gained a placement similar to that in the NMR structure. In Figure 3, the distance between the center of mass (COM) of L3 and the COM of the whole protein is plotted: the plot shows a major deviation of L3 from the whole protein at the beginning, with an abrupt transition around 20 ns. This behavior, along with structure visualization at various stages of the simulation (inset of Figure 3), manifests that L3 docked into the HC at the early stage of the simulation and remained trapped into the HC for the entire duration afterwards; the initial 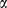-helix conformation of L3 was lost during the settling of the segment into the crevice. A test simulation carried out at higher temperature (350 K) reveals the same mode.

In the first 100 ns of the MD-NMR simulation, the RMSD values are larger than 5 Å: this means that during the early stage of the MD-NMR simulation the protein core remarkably differs from the crystallographic structure. Yet, we observe that L3 entered the HC in this early stage and remained trapped into the crevice for the remainder of the time evolution, as in the MD-XR simulation. Between 100 ns and 300 ns, the RMSD values decrease and reach a local basin between 5 Å and 4 Å. After 300 ns the RMSD values settle a plateau around 2 Å. The RMSD trends attest that the solution structure during the MD-NMR simulation clearly underwent major conformational rearrangements (Table S3) and eventually converged to the crystal structure.

**Figure 4:**
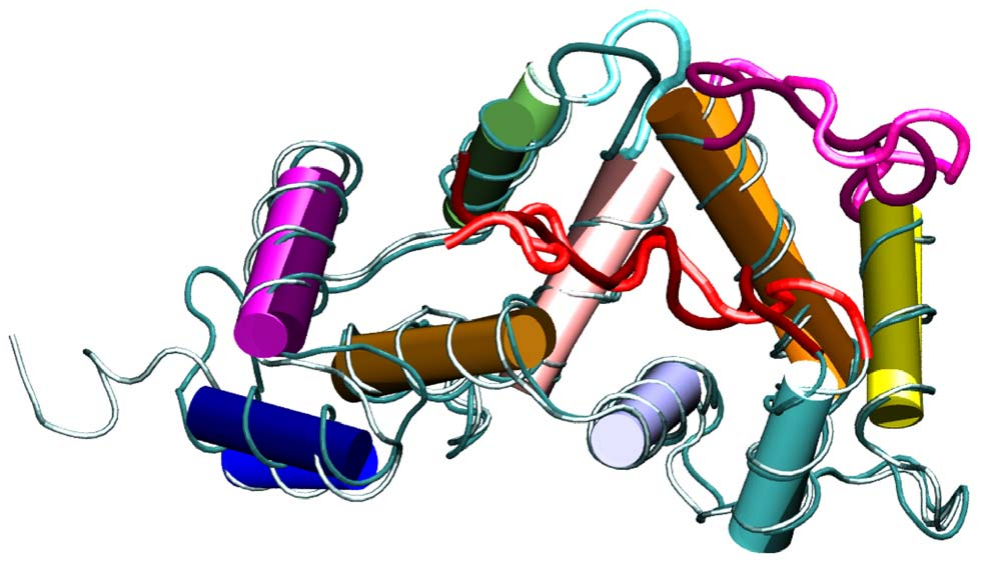
Representative structures of the most populated clusters. The clustering algorithm was applied during the last 100-NMR and MD-XR trajectories are shown in light and dark colors, respectively. The color code for the different protein segments is the same as in Figure 1.

**Figure 5:**
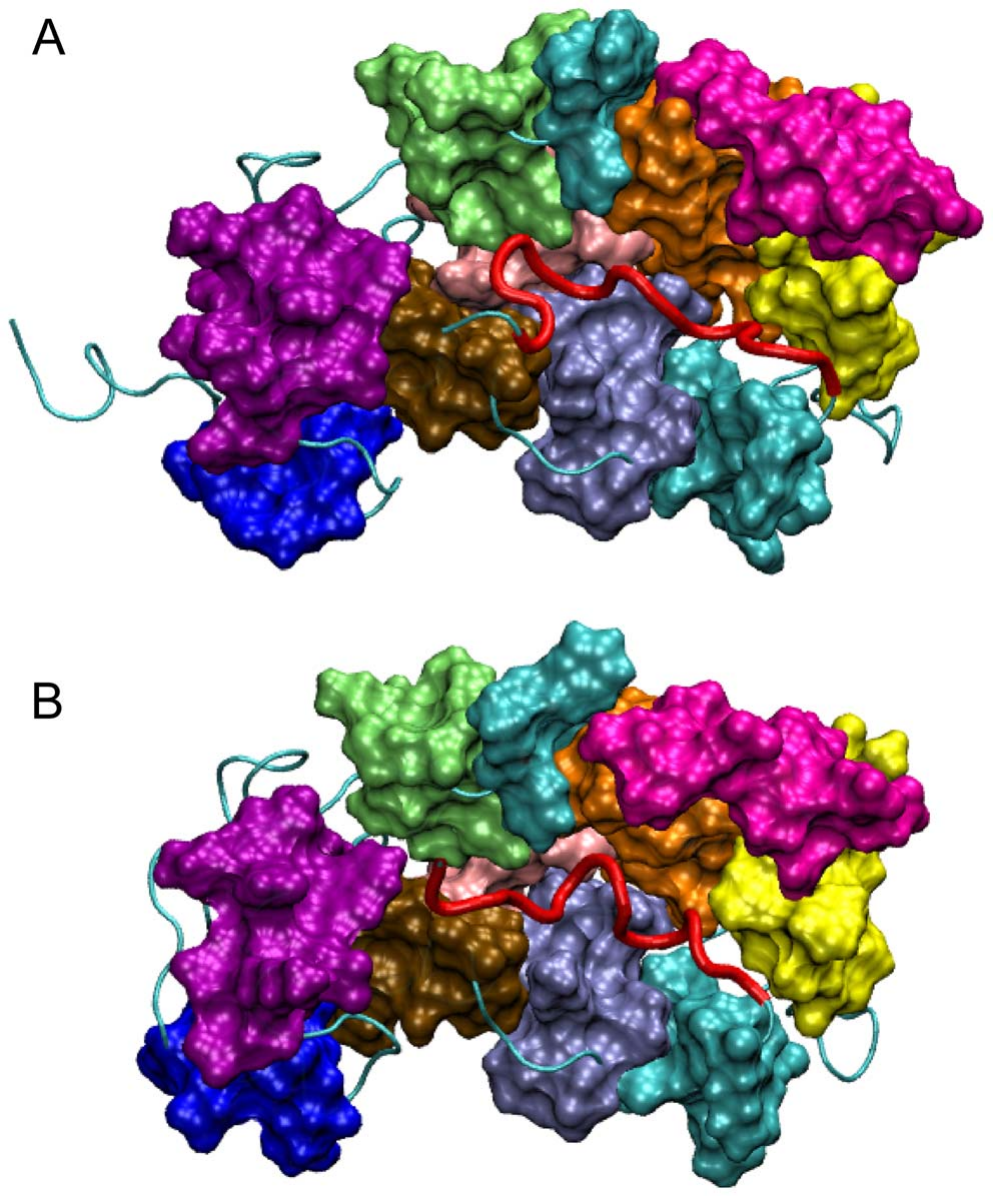
Surface representation of the most representative structures. The surface representation highlights the shape of the HC and the allocation of the L3 into the crevice. (A) Most representative structure for the MD-XR trajectory; (B) most representative structure for the MD-NMR trajectory.

In fact, the RMSD between the average structures evaluated over the last 100 ns of each simulation is 1.9 Å, much smaller than the initial 4.4 Å (Figure S2). Representative structures were extracted from the trajectories by a clustering algorithm applied during the final 100 ns of each simulation (see Materials and Methods). The representative structures of the most populated clusters (also called in the following *most representative structures*) from MD-XR and MD-NMR are visualized in Figure 4. The two structures show a considerable overlap, which is consistent with the low protein-core RMSD value of 2.1 Å between them. Furthermore, Figure 5 shows that the shapes of the HC are equal in these two representative structures, and that L3 is almost totally accommodated in the groove in both cases.

The RMSD values between any two representative structures of all the clusters obtained from the two trajectories span between 1.9 and 2.8 Å (Table S4), demonstrating that protein conformations in the last 100 ns of both simulations belong to the same state. 80% of the NOE restraints evaluated in the last 100 ns of both simulations was respected, showing a good agreement between experimental and simulation data. More interesting is the evolution of the NOEs violation during the MD-NMR simulation. At the early stage of the MD-NMR simulation, namely between 50 and 150 ns the average violation is 2.2 Å. In the last 100 ns of the MD-NMR simulation, the average violation decreases to 1.9 Å. During the MD-XR the average violation is constant, 1.5 Å. These NOE trends are an additional indication that during the MD-NMR simulation the protein core of the NCS-1 relaxed toward the state of the protein core of the MD-XR.

**Figure 6:**
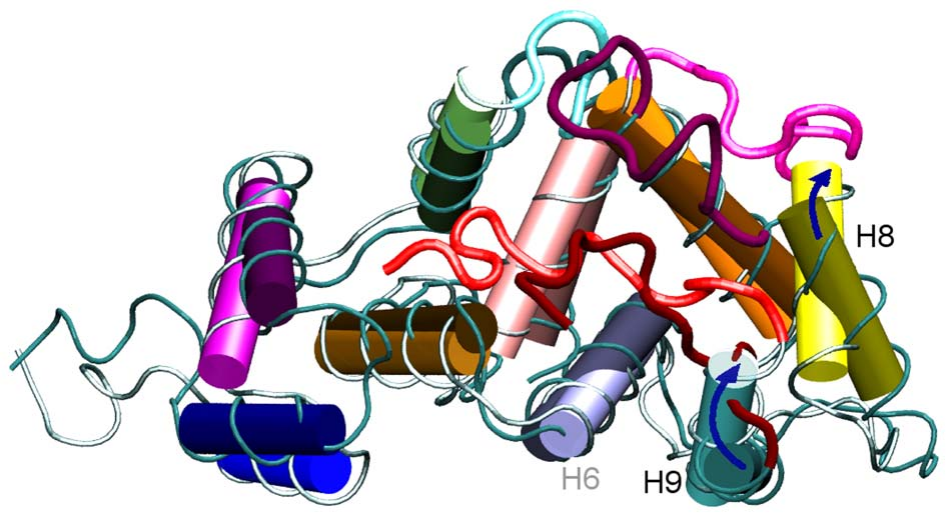
Most representative structure of the MD-NMR trajectory (bright color) and corresponding initial structure (dark color). Blue arrows highlight the movement of H9 and H8 with respect to the NMR initial positions.

To inspect in more detail the evolution of the protein conformation during the MD-NMR simulation, the RMSDs for significant protein regions were calculated (Table 1). Such partial RMSD values were calculated for each separate region between the most representative structure of the MD-NMR trajectory and the corresponding initial structure. The RMSD data reported in Table 1 clearly show that the buried 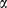-helices H2, H3, H4, H5, H6 and H7, as well as the 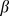-strands involved in the EF motifs, undergo minor rearrangements (small RMSD). As expected, the random coils L3 and L2 have the highest RMSD values. The high RMSD value for L3 is consistent with the docking of L3 into the HC at the early stage of the time evolution, as noted above. The high RMSD value for L2 is due to the high mobility of this segment. The large RMSD value of 7.3 Å for H1 suggests that this segment is very mobile. The H8 and H9 helices also have a fairly large RMSD value of 5 Å. Indeed, these helices underwent an appreciable conformational rearrangement during the dynamics. In particular, during the MD-NMR simulation H9 was “wedged” between H8 and H6, further closing the edge of the HC, whereas H8 was reoriented to extract the L2 segment from the HC (Figure 6).

**Table 1:** Partial RMSDs for the MD-NMR simulation.

Segment	Definition	RMSD(Å)
H1	11–18	7.3
H2	24–34	3.5
H3	45–54	2.8
H4	62–72	2.6
H5	82–93	1.6
H6	98–108	1.8
H7	118–132	1.3
H8	146–155	5.0
H9	166–174	5.0
*β*1	42–44	1.9
*β*2	58–60	3.1
*β*3	79–81	1.8
*β*4	115–117	2.0
*β*5	136–138	11.6
*β*6	163–165	3.3
L1	56–61	3.7
L2	133–145	10.2
L3	175–187	8.3

Partial RMSD evaluated after structure alignment over the backbone atoms of protein core residues. RMSD values for the helices H1 to H9, the 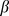-strands 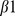 to 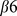, and the loops L1, L2, and L3 between the most representative structure of the MD-NMR trajectory and the corresponding initial structure. 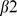 and 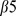 are sub-structures of the L1 and L2 segments respectively.

A test simulation, carried out at 350 K starting from the last structure of the MD-NMR simulation, shows that the L3 segment remains firmly docked into the HC and the PC does not undergo any major conformational changes (Figure S3). These evidences confirm that during the MD-NMR simulation the system converged to a stable state.

To gain insights into the importance of specific residues in stabilizing the NCS-1 structure, pairwise residue-residue potential interaction energies were calculated for both simulations during the respective final 100 ns and then averaged (see Material and Methods). The result of this procedure for each trajectory is the inter-residue energy map (IEM), which reflects the basic interactions that stabilize the native structure of a proteins [27], [28].

**Figure 7:**
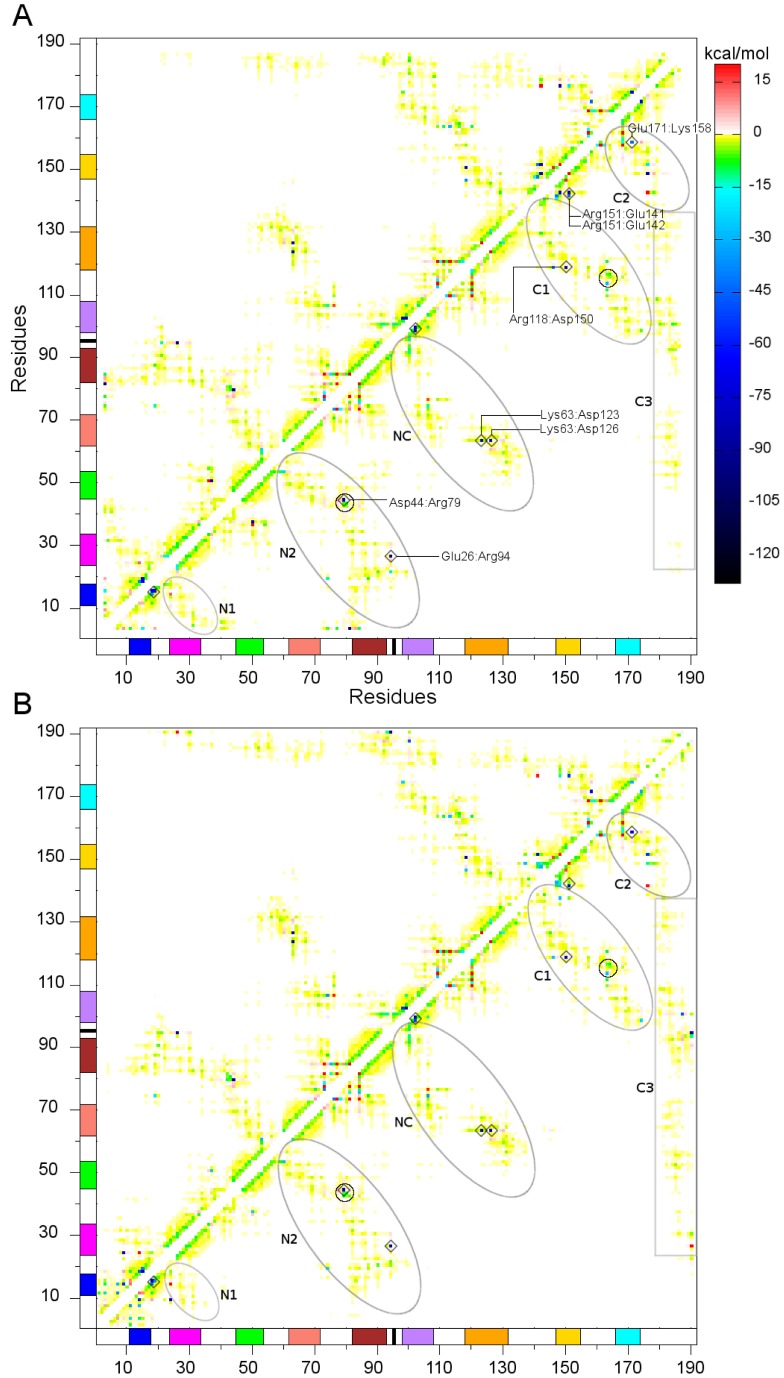
Inter-residues energy maps. (A) IEM for the MD-XR simulation, (B) IEM for the MD-NMR simulation. The interactions among different helix structures are enclosed by ovals. The helix color scheme, consistent with that of Figure 1, is reported along the axes. Interactions between L3 and the PC are highlighted by a rectangle. Small circles in N2 and C1 highlight the 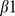-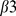 and 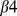-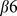 interactions. The conserved salt bridges with energy lower than −30 kcal/mol are marked with diamonds.

The computed IEMs are illustrated in Figure 7. The inter-residue energy values range between −120 kcal/mol and +20 kcal/mol. The strongest attractive interactions are shown in dark-blue (negative values), whereas the most unfavorable interactions (positive values) are shown in red. The yellow and light-green zones span an energy interval between −2 and 0 kcal/mol and are mainly due to weak dispersion and hydrophobic interactions. The green zone spans the interval between −15 and −2 kcal/mol: this range is typical of hydrogen bonds. The cyan and light-blue zones span the interval between −30 and −15 kcal/mol and highlight strong polar interactions or weak ionic interactions. Dark points reflect ionic interactions. The green points that form straight segments on the two sides of the diagonal mainly reflect the 1–4 hydrogen bonds that define the secondary structure of the 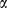-helices. The lack of interactions along the very diagonal is due to the loops (L1, L2 and L3) and to the presence of the calcium binding EF motifs. The calcium ions are coordinated in the binding pocket by negatively charged residues. Because the calcium ions were not considered in the potential energy calculation, only the repulsive interactions between negatively charged residues are reflected in the IEMs.

The two maps in Figure 7 highlight the same interactions and same topology, thus yielding another proof that the two simulations fell into the same stable conformation.

The ovals in Figure 7 marks the interactions that mainly define the topology of the IEMs. The N1 and N2 zones refer to inter-residue interactions between residues of the N-domain. More specifically, the N1 zone embodies the interactions between H1 and H2 (H1-H2), whereas N2 expresses the H3-H5, H1-H5 and H2-H5 interactions. The small circle inside the N2 oval includes the 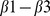 interaction. The NC zone refers to interactions between the N-domain and C-domain that involve the H5-H6, H4-H6 and H4-H7 helix pairs. The C1 and C2 zones refer to the inter-residue interactions between residues of the C-domain. The C1 zone highlights the H6-H9 and H7-H8 interactions and the internal circle in C1 pertains to the 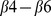 interaction. The C2 zone contains the H9-H8 interaction. The rectangular C3 zone highlights inter-residue interactions between residues of L3 and residues of the PC that shape the HC, and show that the L3 segment interacts with the NCS-1 PC mainly through hydrophobic interactions. In this structural portion the Asp187 residue could interacts with Lys100 or Arg94 than with other residues, eventually anchoring the very terminal tail of the L3 segment to the HC.

Among the various point interactions, we focus our analysis on the strongest and conserved interactions in both IMEs, i.e., the salt bridges (see also the filtered maps in Figures S4 and S5). The conserved salt bridges with energy lower than −30 kcal/mol are marked with diamonds in Figure 7. Let us start our comments from intra-helix salt bridges located in H1 and H6. In H1 the salt bridges are Glu14:Arg18, Glu15:Arg18 and Glu15:Lys19, whereas in the H6 they are Arg102:Glu99 and Arg102:Asp98. It is worth nothing that Asp98 of H6, however, can also interact with the Lys174 residue of H9 to accomplish an inter-helix salt bridge between H6 and H9. The stabilization of the inter- *versus* intra-helix salt bridge involving Asp98 (i.e., Asp98:Lys174 *versus* Asp98:Arg102) relies, therefore, in the possibility of the Arg98 to interact with Arg102 or Lys174. In this sense, we propose that a mutation that consists in replacing Arg102 with an uncharged residue could affect the mobility of H9 and L3, compromising the docking process of L3 into the HC. In fact, it is known that the substitution of Arg102 with glutamine affects the structural and dynamical features of the C-terminal tail, providing a molecular counterpart to the hypothesis that the Arg102Gln mutation is implicated in the autism disease [10], [19], [29].

Other salt bridges exist between residues that belong to distinct protein segments. The conserved Arg151:Glu141 and Arg151:Glu142 bridges, for example, connect L2 to H8. The Arg118:Asp150 bridge connects H7 to H8 and the Glu171:Lys158 bridge connects H8 to H9. The Asp44:Arg79 bridge stabilizes the 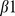-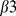 interaction, whereas the Glu26:Arg94 bridge connects H2 to H5. The Glu26 and Arg94 residues interact in an “end-on” manner [21], thus suggesting a key role in the conformational specificity of the N-domain. The Lys63:Asp123 and Lys63:Asp126 bridges involve one residue of the N-Domain and two residues of the C-domain: these two salt bridges can coexist or exist exclusively; they start from Lys63 and, bifurcating towards Asp123/126, are able to connect the H4 helix of the N-domain to the H7 helix of the C-domain.

## Discussion

The biological function of the NCS protein family is related to the ability of interacting with different target proteins and regulating their action. NCS proteins bind target ligands trough a conserved hydrophobic binding pocket, which can differ in shape and size to regulate the target specificity [1], [3], [17], [18], [29]. However, to guarantee the target specificity, each member of the NCS family seems to use multiple regulatory mechanisms. The biological function of recoverin for example is strongly related to the myristoyl switch mechanism induced by the Ca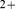 concentration. Weiergräber *et al.* [30] showed that truncating the last 12 basic residues of the C-terminus altered the Ca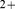 affinity of recoverin, demonstrating that C-terminal tail acts as a “internal modulator of Ca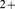 sensitivity”. In particular, they observed that in truncated recoverin (Rc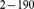, PDB code 2HET), the C-terminal tail is docked in the hydrophobic pocket, resembling the binding mode of L3 to the HC in NCS-1. On the contrary, experimental evidences indicate that the biological function of NCS-1 is not strictly regulated by the Ca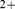 concentration and/or myristoyl switch mechanism as in recoverin [1], [30], corroborating the hypothesis that the hydrophobic nature of the C-terminal tail of NCS-1 is the main regulator of the NCS-1 specificity (in fact, the C-terminal tail of the NCS-1 is hydrophobic, whereas the C-terminal tail of recoverin is hydrophilic [17]).

The molecular dynamics simulations presented here assess a consensus structural state, which is reached from either the NMR solution structure or the X-ray structure, if the PEG molecules are removed from the HC in the latter. The consensus structure has a core similar to the X-ray structure, while the location of the C-terminal inside the HC is akin to the NMR solution structure: we infer that in the experimental crystal phase the consensus state is inhibited by the presence of PEG molecules that occupy the HC. Our results suggest that the L3 location is sensitive to the environmental condition and in the absence of a substrate it has the role of stabilizing the protein fold by segregating the HC from water. These findings are in agreement with NMR experimental evidences [19] and confirm the hypothesis that the C-terminal residues occupy the hydrophobic crevice as a ligand mimic. Our simulations also grant a dynamical description of the docking of L3 in the HC: L3 and HC interact mainly by hydrophobic interactions, eventually stabilized by salt-bridge interactions. The competition between the intra-molecular ligand mimic L3 and the proper inter-molecular protein target, therefore, could establish a dynamical mechanism to modulate the target specificity of NCS-1. Moreover, the affinity of L3 towards the HC could facilitate the extrusion of the myristoyl from the HC during the membrane binding and prevent the binding of the myristoyl chain to the HC.

The analysis of inter-residue interaction maps has evidenced the presence of various salt bridges that connect different secondary structure elements, thus contributing to determine the protein tertiary structure. Assigning a biological function to an individual salt bridge is a controversial issue. However, it is a widespread idea that the stability of the salt bridges is sensitive to the environment and that they have an important role in stabilizing/destabilizing protein structures [20]–[22], [31], [32]. Therefore, the identified salt bridges define residues whose mutation may lead to profound effects on the NCS-1 stability and biological function. In particular, we have remarked the salt-bridge network involving Arg98, Arg102 and Lys174, which could affect the mobility of H9 and L3, providing a possible rationalization on how the Arg102Gln mutation could be connected to the autism disease [10].

## Materials and Methods

### NCS-1 structure

In Figure 1A the cartoon representation of the crystallographic protein structure of unmyristoilated NCS-1 (PDB code 1G8I) [4] is shown. Crystallographic water and residual chain glycols located in the HC were removed and the HC is left empty. In Figure 1B the NMR structure (model 1 in PDB 2LCP) [19] is shown. The NCS-1 structure mainly consists of nine 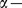
helices arranged in four EF hands. The latter are organized in an N-terminal pair (EF1 and EF2) and a C-terminal pair (EF3 and EF4); as shown in Figure 1 only EF2, EF3 and EF4 are able to bind Ca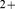 ions. The N-terminal domain (ND) and C-terminal domain (CD) are linked by a hinge loop (HL) consisting of residues S93 to D98. Adopting the nomenclature of Heidarsson *et al.* [19], the nine helices are H1 (E11-R18), H2 (E24-F34), H3 (A45-Q54), H4 (T62-F72), H5 (F82-S93), H6 (D98-Y108), H7 (R118-V132), H8 (Q146-M155), H9 (L166-K174). Six 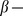
type conformations, namely 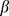
1 (Q42-D44), 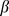
2 (F58-D60), 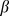
3 (R79-V81), 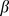
4 (Y115-T117), 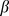
5 (V136-L138) and 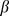
6 (K163-T165), are present in the structure. Four of them, i.e. 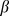
1, 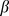
3, 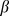
4 and 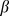
6, are involved in the calcium binding loops (i.e., the EFs), whereas 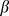
2 is included in the short loop L1 (F56-P61) and 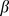
5 is included in the long loop L2 (G133-P145). The eight helices H2-H9 shape the HC at the opposite side of the EF hand motifs. H4, H5 and H6 shape the floor of the crevice. H3 and H7 in one side and H9 in the other side shape the long walls of the HC, whereas H8 and H2 close the HC at the opposite edges (see Figure 1). L1 and L2 connect H3 and H4 with H7 and H8, respectively, whereas a third loop (L3, consisting of residues D176-D190) shapes the C-terminal tail.

### Molecular dynamics simulations

Molecular dynamics simulations were performed with NAMD (v2.8) [33]. The system preparation was done with VMD (v1.9.1) [34]. The CHARMM27 force field [35] was used for the protein, calcium ions and the counterions, whereas the TIP3P [36] force filed was used for water.

Two different experimental structures were used as the starting points for two independent MD simulations of the NCS-1 protein. One simulation started from the crystal structure [4], chain B of the PDB 1G8I, after removing all crystallographic waters and residual chain glycols located in the HC. Another simulation started from the first of the NMR structures deposited by Heidarsson *et al.* [19], PDB code 2LCP. The simulations were conducted using periodic boundary conditions (PBC). The water layer separating the protein from any of its periodic images was at least 14 Å thick in each simulation box. To guarantee neutrality 3 Na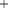 ions were placed randomly at a distance larger than 5 Å from the protein. The bonds between hydrogen and heavy atoms were constrained with SHAKE [37]. The r-RESPA multiple time step method [38] was employed with 2 fs for bonded potentials, 2 fs for the short-range part of non-bonded potentials, and 4 fs for the long-range part of the non-bonded potentials [33]. The long-range part of the electrostatic interaction was treated with the Particle-Mesh-Ewald (PME) method [39]. The distance cut off for non-bonded interactions was set to 12 Å, and a switching function was applied to smooth interactions between 10 and 12 Å.

All simulations were conducted in the NPT ensemble. The temperature was set to 310 K and regulated via a Langevin thermostat [40]; the pressure was set to 1 atm and regulated via an isotropic Langevin piston manostat [41].

Each simulation was preceded by a preparation phase consisting of both geometry optimization and constrained MD. 2000 steps of conjugate gradient geometry optimization were initially conducted with harmonic restraints on the protein heavy atoms using a force constant of 1 kcal/(mol Å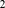). After energy minimization, the system was simulated for 1 ns: in the first 0.5 ns harmonic restraints were applied on the protein heavy atoms with a force constant of 1 kcal/(molÅ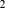); in the last 0.5 ns the force constant was scaled to 0.1 kcal/(mol Å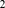). Subsequently 25000 steps of minimization without restraints were performed. The final structures were then used as the distinct initial conditions for unrestrained molecular dynamics.

The MD-XR simulation that started from the X-ray structure was 250 ns long. The MD-NMR simulation that started from the NMR structure was 525 ns long. Two further test simulations starting from the crystal structure and from the last structure of the MD-NMR simulation were conducted at 350 K; both simulations were carried out for 50 ns.

### Analysis

Root mean square deviations (RMSD) were evaluated after the structure alignment of the backbone residues 11 to 174, the protein core.

Cluster analysis was performed with WORDOM [42], by comparing the RMSD values for the PC calculated at every 50 ps over the last 100 ns of the MD simulation. The cut off was set equal to 2 Å and clustering was accomplished by using a hierarchical algorithm.

Nuclear overhauser enhancemnt (NOE) interproton distances were calculated from the simulation and compared to the experimental data [19]. Due to the presence of multiple equivalent proton definitions (e.g., interproton distances between two methyl groups), an effective interproton distance was evaluated by summing each distance between pairs of proton weighted by the sixth power; interproton distances were evaluted for each structure and averaged over time by the sixth power [43]. A NOE distance was considered violated if it exceeds the upper distance limit increased by 0.6 Å; each distance violation (Vij) was defined as difference between the NOE distance and the upper limit, whereas the average violation 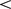
V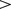 was evaluated by averaging all the Vij values over the number of total violated NOEs.

Inter-residue interaction potential energies for each pair of residues 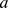 and 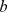 were evaluated. The long-range part of the electrostatic interactions was neglected and the interaction energy between residues with indexes 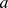 and 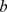 was calculated when the condition 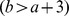 was satisfied. The calcium ions were not included in the calculation. To account for the fluctuations of the protein, the pair interaction energies were calculated every 50 ps over the last 100 ns of the MD simulation and averaged over the time.

## Supporting Information

Figure S1
**Comparison between the most representative MD-XR structure (light color) and the crystallographic structure 1G8I (dark color).** The color code that distinguishes the various protein segments is the same as used in the manuscript. The most representative structure pertains to the final 100 ns of the MD-XR simulations. Alignment was performed over backbone atoms of residues 11 to 174. The aligned structures do not show particular structural differences in the orientation of the 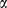-helices. The most significant difference concerns the location of the L3 segment. L3 is external to the HC in the crystal structure (used as the starting point of the MD-XR dynamics), whereas it is docked in the HC in the most representative dynamical structure. See Table S2 for more details.(TIF)

Figure S2 **Average structures calculated over the last 100** **ns of the two MD simulations.** Blue: average backbone structure for the MD-XR simulation. Red: average backbone structure for the MD-NMR simulation. Alignment was performed over backbone atoms of residues 11 to 174.
(TIF)

Figure S3 **Comparison between the initial (dark color) and final (light color) structures of the MD-NMR simulation at 350 K**. The color code that distinguishes the various protein segments is the same as used in the manuscript. Alignment was performed over backbone atoms of residues 11 to 174. This comparison does not reveal any significant structural differences.
(TIF)

Figure S4 **Energy-filtered IEM for the MD-XR trajectory.** Energy lower than −30 kcal/mol are shown in the map, which thus highlights only the locations of salt bridges.
(TIF)

Figure S5 **Energy-filtered IEM for the MD-NMR trajectory.** Energy lower than −30 kcal/mol are shown in the map, which thus highlights only the locations of salt bridges.
(TIF)

Table S1
**NMR vs X-ray structures: partial RMSDs.** Backbone RMSDs between the crystal structure (segment B of the PDB file 1G8I) and the solution structure (first structure in the PDB file 2LCP). Alignment was performed over backbone atoms of residues 11 to 174. Data clearly show that the buried 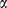-helices H3, H4, H5, H6 and H7 have small RMSD values, whereas the loops L2 and L3 and the strand 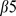 have the highest RMSD values. The fragments L1 and 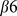 have intermediate RMSD values of 3.2 Å and 3.9 Å respectively. The helices H1, H2, H8 and H9 have conspicuous RMSD values spanning from 4.0 Å for H9 to 6.4 Å for H1.(TIFF)

Table S2 **Partial RMSDs evaluted between the representative structure of the most populated cluster of the MD-XR and the crystallographic structure.** The partial RMSDs clearly show that the initial crystallographic structure and the representative structure of the most populated cluster obtained from MD-XR simulation do not differ. Alignment was performed over backbone atoms of residues 11 to 174.
(TIFF)

Table S3 **Partial RMSDs in Å evaluated between the most representative MD-NMR structure and experimental NMR structures.** The labels S1-S6 mark the first six structures contained in the pdb file 2LCP. The partial RMSDs clearly show that the most representative MD-NMR structure differs substantially from the pdb structures, not only in the mobile loops but also in some helices and strands. This is true not only for the structure S1 that we used as a starting point for the MD-NMR run, but also for other deposited structures, meaning that our results and conclusions do not depend on the arbitrary initial condition of the MD-NMR simulation. Alignment was performed over backbone atoms of residues 11 to 174.
(TIFF)

Table S4 **Backbone RMSDs between representative structures from the MD-XR and MD-NMR trajectories.** Cluster analysis was performed in the last 100 ns of the MD-NMR and MD-XR trajectories: 10 clusters were found for MD-NMR and 3 clusters were found for MD-XR. Each cluster contains similar structures and is represented by one such structure. RMSD values are in Å. The order of the clusters is related to the population, namely the number of snapshots that it contains: cluster “1” is the most populated. The values in this Table clearly show a high similarity between the representative structures of both trajectories during the final 100 ns when the consensus status has been attained. Alignment was performed over backbone atoms of residues 11 to 174.
(TIFF)
