# Supplementary figures and images for: The Structure of Neuronal Calcium Sensor-1 in Solution Revealed by Molecular Dynamics Simulations
Source: PLoS One. 2013 Sep 30;8(9):e74383. doi: 10.1371/journal.pone.0074383 (PMC3787052; doi:10.1371/journal.pone.0074383)

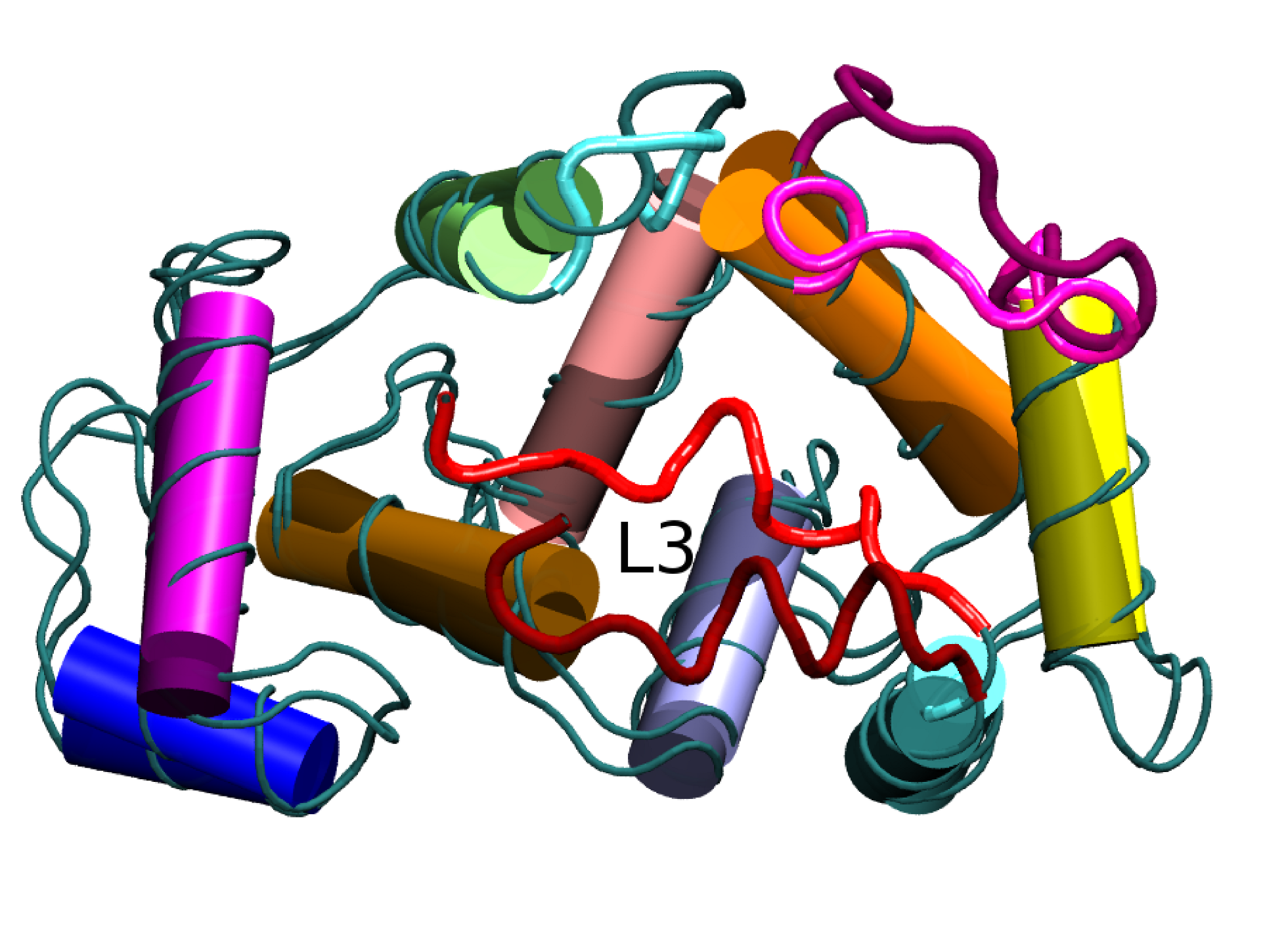

Supplement: Figure S1 — Comparison between the most representative MD-XR structure (light color) and the crystallographic structure 1G8I (dark color). The color code that distinguishes the various protein segments is the same as used in the manuscript. The most representative structure pertains to the final 100 ns of the MD-XR simulations. Alignment was performed over backbone atoms of residues 11 to 174. The aligned structures do not show particular structural differences in the orientation of the -helices. The most significant difference concerns the location of the L3 segment. L3 is external to the HC in the crystal structure (used as the starting point of the MD-XR dynamics), whereas it is docked in the HC in the most representative dynamical structure. See Table S2 for more details. (TIF) [file pone.0074383.s001.tif]

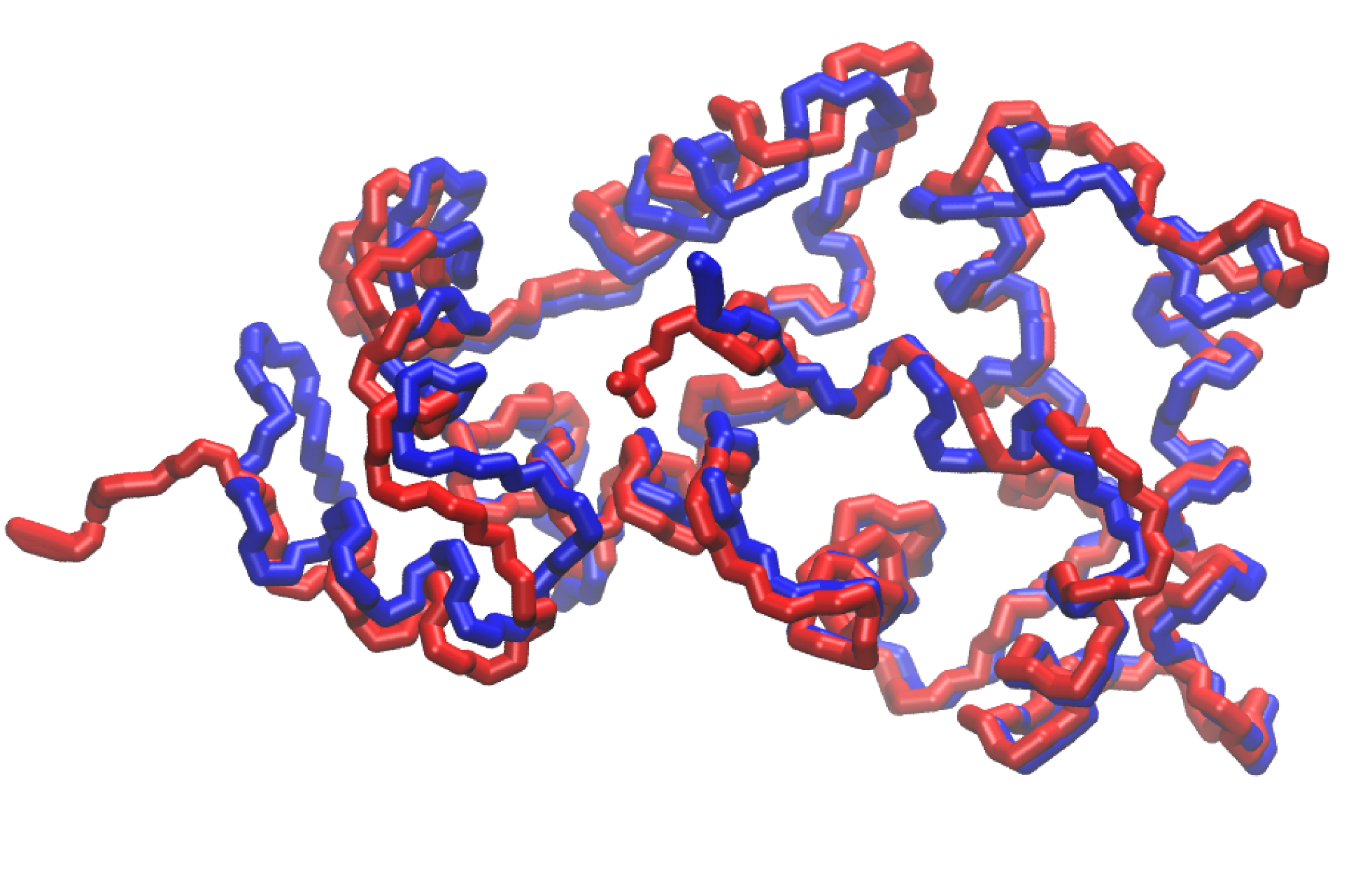

Supplement: Figure S2 — Average structures calculated over the last 100 ns of the two MD simulations. Blue: average backbone structure for the MD-XR simulation. Red: average backbone structure for the MD-NMR simulation. Alignment was performed over backbone atoms of residues 11 to 174. (TIF) [file pone.0074383.s002.tif]

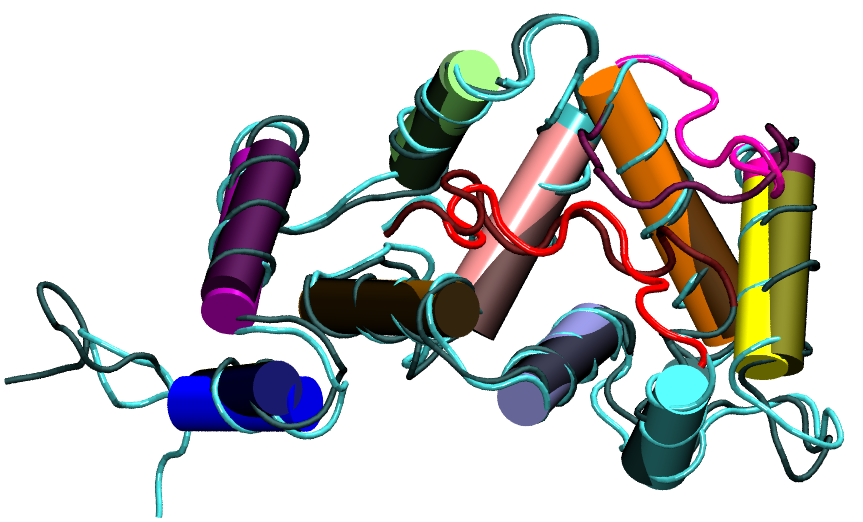

Supplement: Figure S3 — Comparison between the initial (dark color) and final (light color) structures of the MD-NMR simulation at 350 K. The color code that distinguishes the various protein segments is the same as used in the manuscript. Alignment was performed over backbone atoms of residues 11 to 174. This comparison does not reveal any significant structural differences. (TIF) [file pone.0074383.s003.tif]

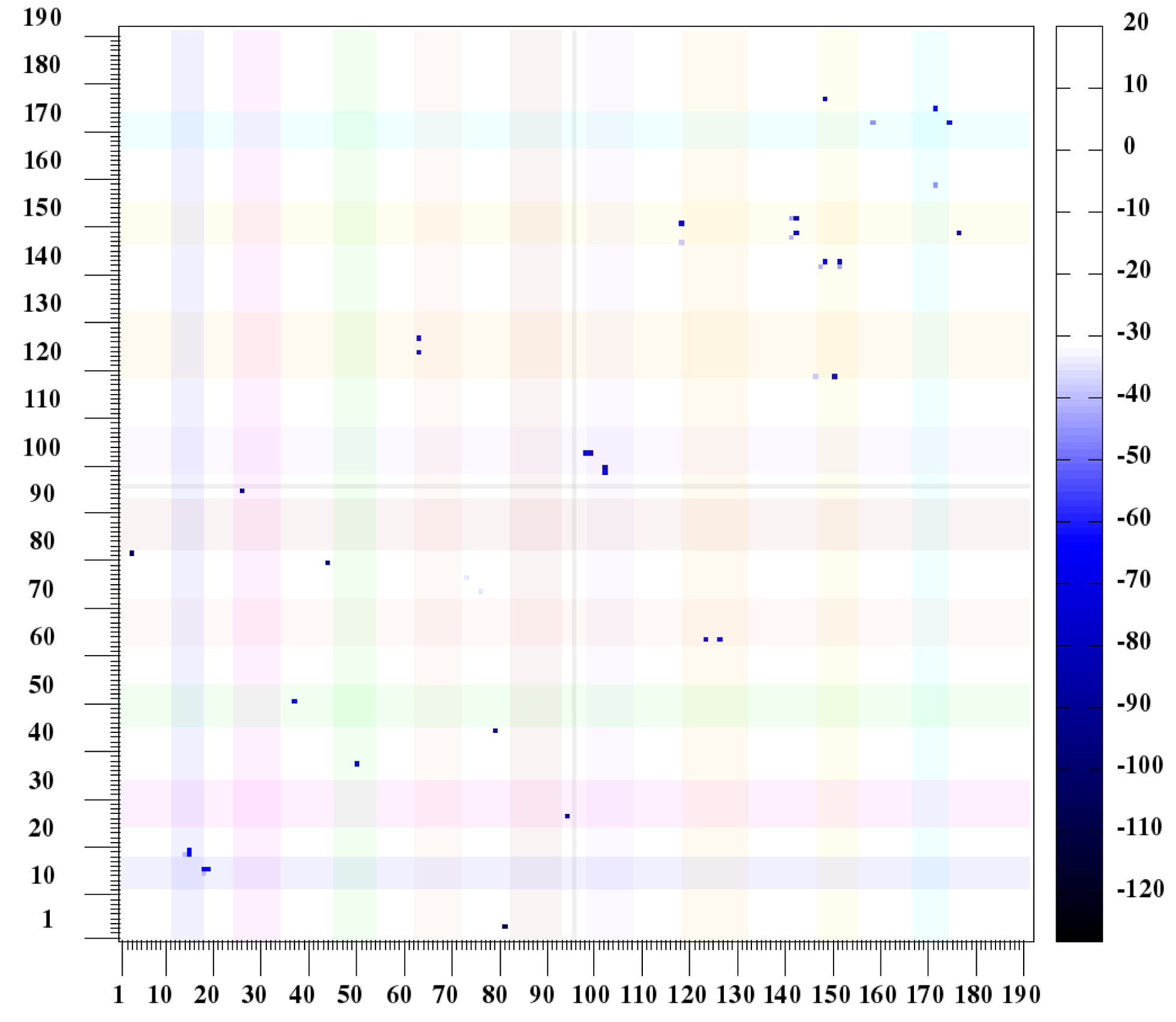

Supplement: Figure S4 — Energy-filtered IEM for the MD-XR trajectory. Energy lower than −30 kcal/mol are shown in the map, which thus highlights only the locations of salt bridges. (TIF) [file pone.0074383.s004.tif]

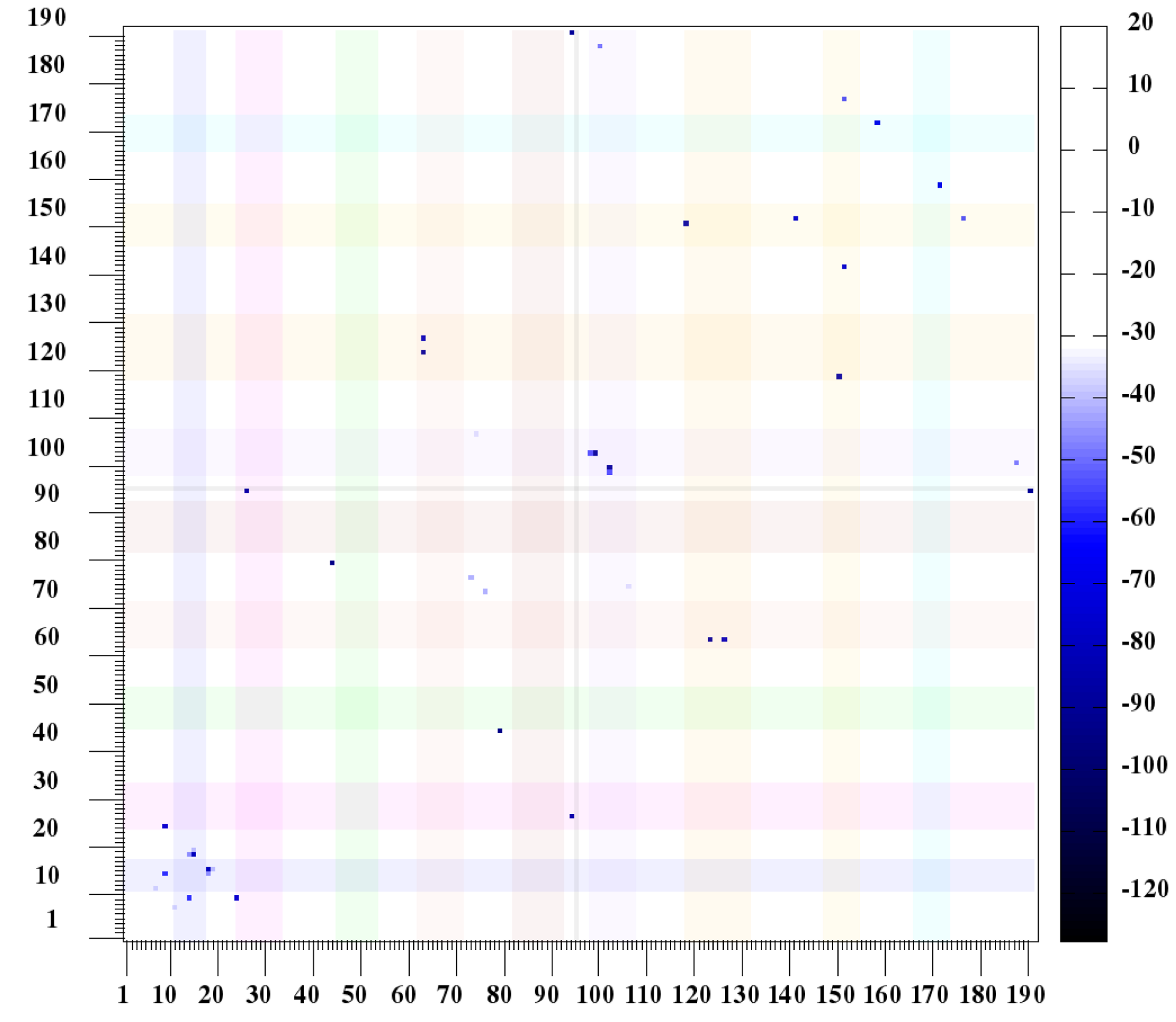

Supplement: Figure S5 — Energy-filtered IEM for the MD-NMR trajectory. Energy lower than −30 kcal/mol are shown in the map, which thus highlights only the locations of salt bridges. (TIF) [file pone.0074383.s005.tif]

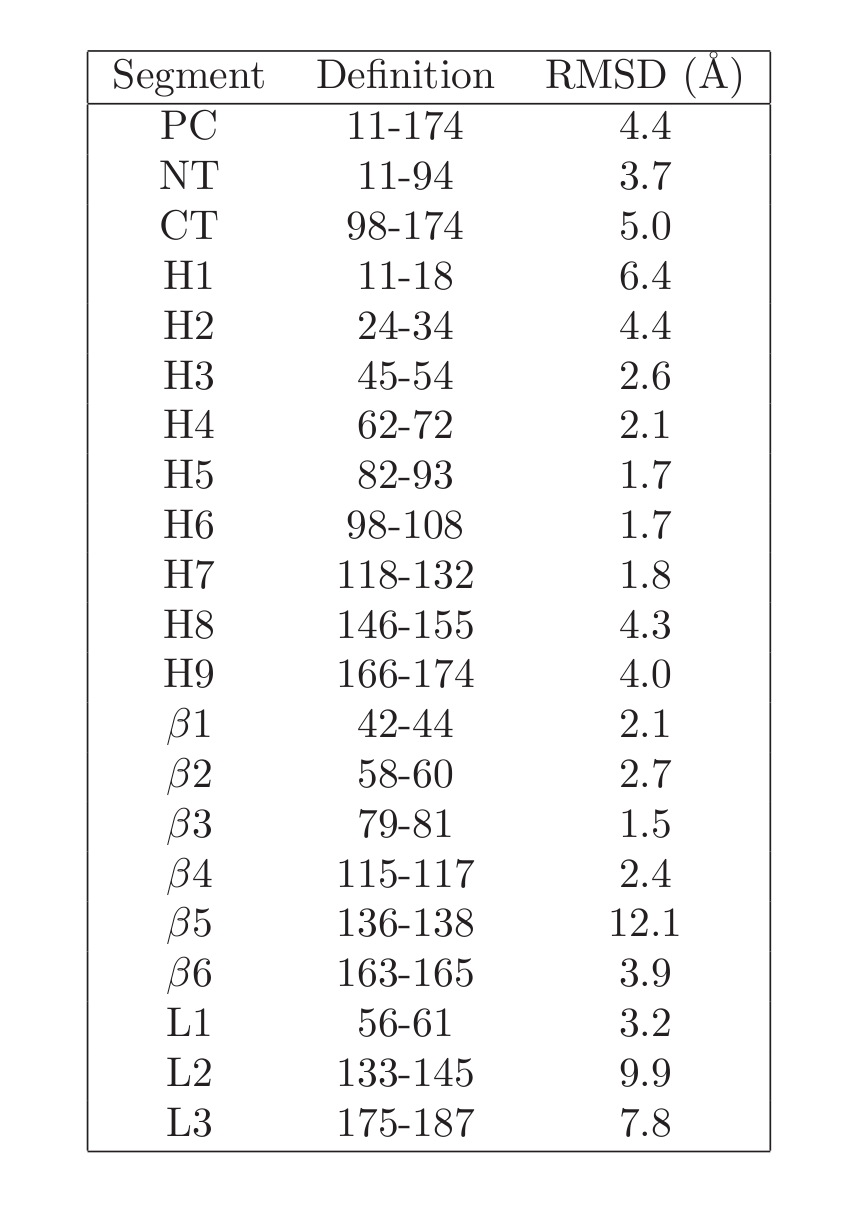

Supplement: Table S1 — NMR vs X-ray structures: partial RMSDs. Backbone RMSDs between the crystal structure (segment B of the PDB file 1G8I) and the solution structure (first structure in the PDB file 2LCP). Alignment was performed over backbone atoms of residues 11 to 174. Data clearly show that the buried -helices H3, H4, H5, H6 and H7 have small RMSD values, whereas the loops L2 and L3 and the strand have the highest RMSD values. The fragments L1 and have intermediate RMSD values of 3.2 Å and 3.9 Å respectively. The helices H1, H2, H8 and H9 have conspicuous RMSD values spanning from 4.0 Å for H9 to 6.4 Å for H1. (TIFF) [file pone.0074383.s006.tif]

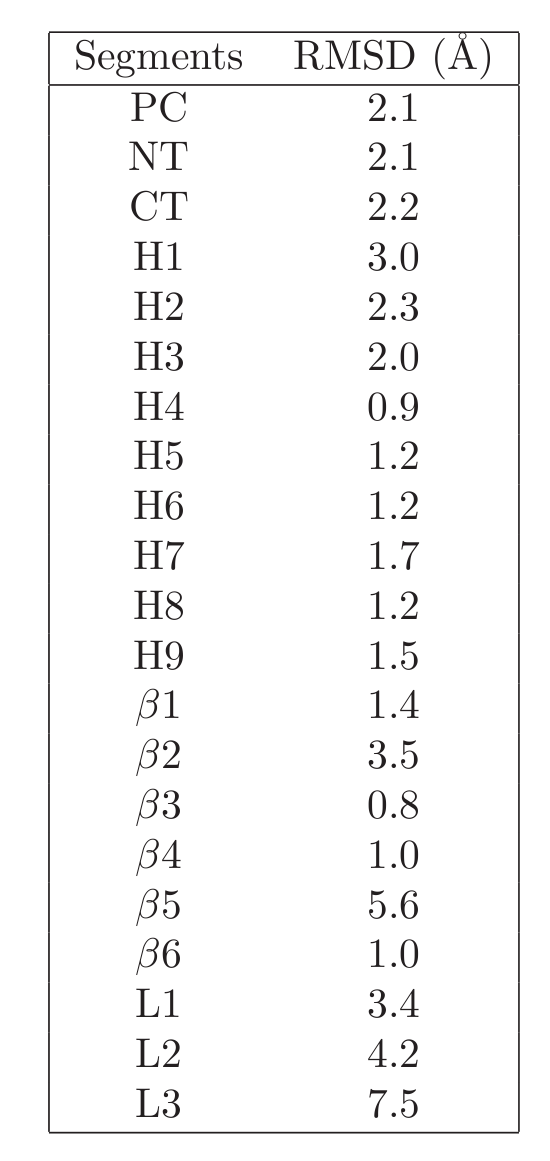

Supplement: Table S2 — Partial RMSDs evaluted between the representative structure of the most populated cluster of the MD-XR and the crystallographic structure. The partial RMSDs clearly show that the initial crystallographic structure and the representative structure of the most populated cluster obtained from MD-XR simulation do not differ. Alignment was performed over backbone atoms of residues 11 to 174. (TIFF) [file pone.0074383.s007.tiff]

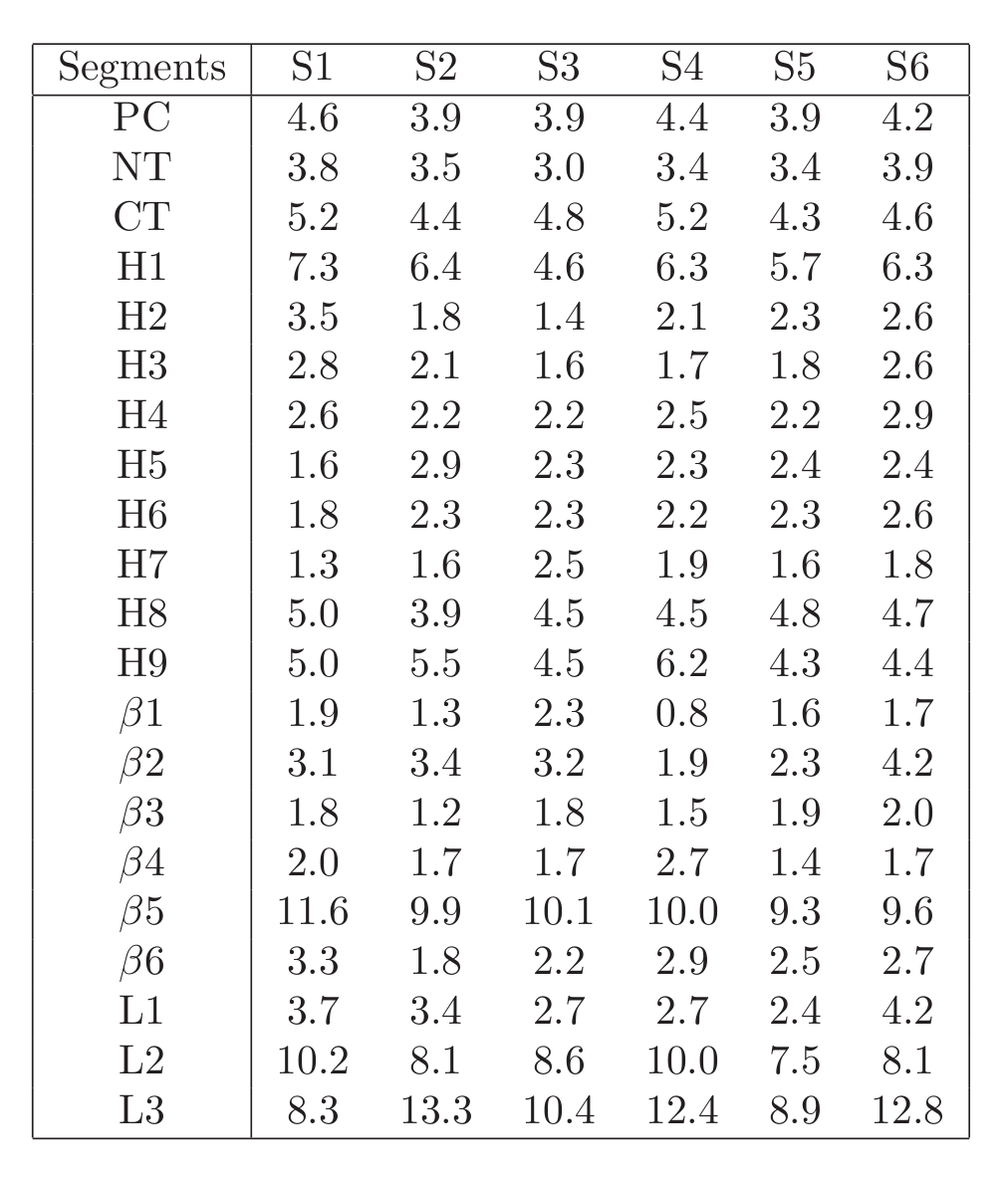

Supplement: Table S3 — Partial RMSDs in Å evaluated between the most representative MD-NMR structure and experimental NMR structures. The labels S1-S6 mark the first six structures contained in the pdb file 2LCP. The partial RMSDs clearly show that the most representative MD-NMR structure differs substantially from the pdb structures, not only in the mobile loops but also in some helices and strands. This is true not only for the structure S1 that we used as a starting point for the MD-NMR run, but also for other deposited structures, meaning that our results and conclusions do not depend on the arbitrary initial condition of the MD-NMR simulation. Alignment was performed over backbone atoms of residues 11 to 174. (TIFF) [file pone.0074383.s008.tiff]

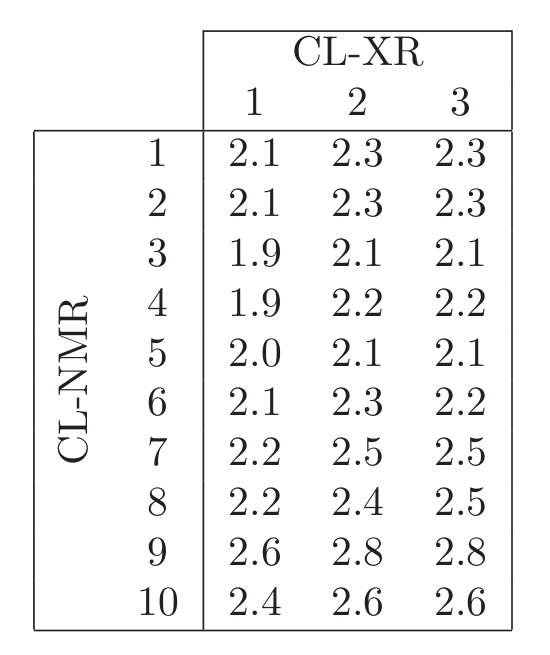

Supplement: Table S4 — Backbone RMSDs between representative structures from the MD-XR and MD-NMR trajectories. Cluster analysis was performed in the last 100 ns of the MD-NMR and MD-XR trajectories: 10 clusters were found for MD-NMR and 3 clusters were found for MD-XR. Each cluster contains similar structures and is represented by one such structure. RMSD values are in Å. The order of the clusters is related to the population, namely the number of snapshots that it contains: cluster “1” is the most populated. The values in this Table clearly show a high similarity between the representative structures of both trajectories during the final 100 ns when the consensus status has been attained. Alignment was performed over backbone atoms of residues 11 to 174. (TIFF) [file pone.0074383.s009.tiff]
